# Polyphyly of Lichen-cryptic Dagger Moths: synonymy of *Agriopodes* Hampson and description of a new basal acronictine genus, *Chloronycta*, gen. n. (Lepidoptera, Noctuidae)

**DOI:** 10.3897/zookeys.421.7424

**Published:** 2014-06-27

**Authors:** B. Christian Schmidt, David L. Wagner, Brigette V. Zacharczenko, Reza Zahiri, Gary G. Anweiler

**Affiliations:** 1 Canadian Food Inspection Agency, Canadian National Collection of Insects, Arachnids, and Nematodes, K.W. Neatby Bldg., 960 Carling Ave., Ottawa, ON, Canada K1A 0C6; 2 Department of Ecology and Evolutionary Biology, University of Connecticut, Storrs, CT 06269; 3 Biodiversity Institute of Ontario, University of Guelph, Guelph, Ontario, Canada; 4 E.H. Strickland Entomological Museum, 218 Earth Sciences Building, Department of Biological Sciences, University of Alberta, Edmonton, Alberta, Canada, T6G 2E9

**Keywords:** *Agriopodes fallax*, *Agriopodes geminata*, *Agriopodes tybo*, dagger moths, *Fraxinus*, Arizona

## Abstract

The taxonomic composition and systematic position of *Agriopodes* Hampson is examined through an integrated approach using adult and larval morphology, biology, and molecular sequence data. The type-species of *Agriopodes*, *Moma fallax* Herrich-Schäffer is shown to be derived within the *Acronicta grisea* Walker species-group; accordingly, *Agriopodes* is relegated to synonymy under *Acronicta* Ochsenheimer, **syn. n.** (Acronictinae). Additionally, molecular markers and morphology show that *Agriopodes* is not monophyletic: *Agriopodes tybo* (Barnes) is not closely related to *A. fallax* nor to *Acronicta*, and is transferred to a new genus, *Chloronycta* Schmidt & Anweiler, **gen. n.** The immature stages of *Chloronycta tybo* **comb. n.** are described and illustrated for the first time. Although previously treated as a valid species, we show that *Agriopodes geminata* (Smith) represents the northern terminus of clinal variation in wing pattern of *A. fallax* and synonymize *A. geminata* under *A. fallax* (**syn. n.**). The history and identity of *Agriopodes corticosa* (Boisduval), a *nomen dubium*, is discussed.

## Introduction

The New World genus *Agriopodes* Hampson (Noctuidae, Acronictinae) has included as many as seven species, united by their striking lichen-mimicking colours of green, white and black. As we show here, their similarity in colour pattern is convergent, and represents a common evolutionary trajectory repeated multiple times across the Noctuoidea. The superficial nature of their shared pattern elements is reflected in the taxonomic history of *Agriopodes*, with species previously removed from the genus now distributed among three different subfamilies outside the Acronictinae (Lafontaine and Schmidt 2010). When Hampson (1909) described *Agriopodes*, he included four species, *Agriopodes fallax* (Herrich-Schäffer), *Agriopodes geminata* (Smith), *Agriopodes tybo* (Barnes) and *Agriopodes viridata* (Harvey). As was typical of taxonomic works of Hampson’s, his generic diagnosis relied on external characters such as wing venation and pattern, scale vestiture, palpi and antennal structure; at the subfamily level within the Noctuidae, these characters can be misleading phylogenetically (Kitching and Rawlins 1998; Fibiger and Lafontaine 2005). Genitalic dissections were not studied by Hampson and his contemporaries as this methodology was still in its infancy.

Barnes and McDunnough (1917) added three more species to *Agriopodes*: *Agriopodes lepidula* (Grote), *Agriopodes teratophora* (Herrich-Schäffer) and *Agriopodes corticosa* (Guenée), an arrangement maintained by McDunnough (1938), except that *Agriopodes lepidula* was transferred to *Leuconycta* Hampson (now Condicinae). Franclemont and Todd (1983) retained *fallax*, *geminata*, *tybo* and *teratophora* in *Agriopodes*; transferred *viridata* to *Cryphia* Hübner (now Bryophilinae); and treated *Agriopodes corticosa* as a non-North American species of unknown identity. Franclemont and Todd (1983) apparently overlooked Forbes’ (1954) transfer of *teratophora* to *Erastria* Ochsenheimer (now *Anterastria* Sugi in Noctuinae). Poole’s (1989) global Noctuidae catalogue maintained Franclemont and Todd’s concept of *Agriopodes* but also included *jucundella* Dyar, which had been transferred to *Cryphia* by Ferguson (1988) the previous year. Presently, this species is placed as “*Elaphria*” *jucundella* in Noctuinae, Elaphriini (Lafontaine and Schmidt 2010).

As part of an ongoing revision of the North American Acronictinae (Schmidt and Anweiler in prep.), we examined the four remaining species of *Agriopodes* (*fallax*, *geminata*, *tybo* and *corticosa*), and it soon became evident that the monophyly of *Agriopodes* was still problematic. Forbes (1954) expressed doubt that *Agriopodes fallax*, the type-species of *Agriopodes*, was distinct from *Acronicta*. He stated that it was “hardly distinguishable...[and] not really distinct from *Apatela* [=*Acronicta*],” but retained *Agriopodes* as a valid genus based on the mesothoracic scale tufts and green colouration of the adult, both characters that appear in other *Acronicta* but to lesser degrees. The larval habitus of *Agriopodes fallax* is not immediately recognizable as belonging to a particular group of *Acronicta* but is certainly within the range of morphological variation encompassed by the genus (see Wagner et al. 2011). The species status of *Agriopodes geminata* poses another problem, being very similar to *Agriopodes fallax*, but thought to occur as a disjunct population in Manitoba and Saskatchewan beyond the eastern North American range of *Agriopodes fallax*. *Agriopodes tybo*, a Sonoran species reaching the U.S. in southeastern Arizona, is shown below to be unrelated to *Agriopodes fallax*, and we propose a new genus for it and describe the immature stages. Lastly, we review the status of *Bryophila corticosa* Guenée, purportedly described from North America and placed in *Agriopodes* by McDunnough (1938).

## Methods and materials

*Morphology.* Adult genitalia were prepared using standard methods, described in detail by Lafontaine (2004). Cleaned, stained genitalia were stored and examined in 30% ethanol and slide-mounted in Euparal before being photographed. As Acronictinae, Pantheinae, Balsinae and Raphiinae (the latter included in Dilobinae by Fibiger et al. 2009) are thought to be closely related, we examined the external and genitalic morphology of nearly all New World species presently included in these subfamilies, the vast majority of which are North American (Poole 1989; Lafontaine and Schmidt 2010). We also examined representative species of European and Asian taxa, including type-species of all European (Fibiger et al. 2009) and most Asian acronictine genera (Holloway 2011; Inoue et al. 1982; Kononenko and Han 2007).

*Molecular analysis.* We compared molecular variation of *Agriopodes fallax* to other Acronictinae using eight gene regions, namely *cytochrome c oxidase subunit 1* (COI) (1477 bp) from the mitochondrial genome and *elongation factor-1* α (EF-1α) (1240 bp), *ribosomal protein S5* (RpS5) (617 bp), *carbamoylphosphate synthase domain protein* (CAD) (859 bp), *cytosolic malate dehydrogenase* (MDH) (407 bp), *glyceraldehyde-3-phosphate dehydrogenase* (GAPDH) (691 bp), *isocitrate dehydrogenase* (IDH) (716 bp) and *wingless* (400 bp) genes from the nuclear genome. All genes are single-copy, protein-coding exons and have previously been found to be highly informative in phylogenetic analyses of Lepidoptera at multiple taxonomic levels (Zahiri et al. 2011, 2012, 2013a, b). Voucher data for DNA samples are given in Table 1.

**Table 1.** Specimen voucher data and GenBank accession numbers for samples used in phylogenetic analysis. Dash indicates DNA markers that did not amplify.

Code	Genus Species	Country	CAD	COI-BEGIN	COI-END	EF1A-BEGIN	EF1A-END	GAPDH	IDH	MDH	RPS5	WINGLESS
**MM06745**	*Craniophora ligustri*	FINLAND	HQ006948	HQ006148	HQ006855	HQ006246	HQ006341	HQ006432	HQ006498	HQ006577	HQ006665	HQ006757
**RZ619**	*Harrisimemna trisignata*	USA	–	KC819665	KC819683	KC819699	KC819716	KC819732	KC819747	KC819762	KC819779	–
**RZ620**	*Polygrammate hebraeicum*	USA	KC819652	KC819666	KC819684	KC819700	KC819717	KC819733	KC819748	KC819763	KC819780	KC819797
**RZ607**	*Acronicta modica*	USA	–	KJ726386	KJ726386	KJ726393	KJ726393	KJ726400	KJ726407	KJ726416	KJ726419	KJ726432
**RZ597**	*Acronicta americana*	USA	–	KC819662	KC819680	KC819696	KC819713	KC819729	KC819744	KC819760	KC819776	KC819794
**MM01529**	*Acronicta rumicis*	FINLAND	GU828163	GU828666	GU828464	GU828997	GU829280	GU829792	GU830053	GU830372	GU830662	GU829551
**RZ602**	*Acronicta impleta*	USA	KJ726383	KJ726387	KJ726387	KJ726399	KJ726399	KJ726401	–	KJ726417	KJ726423	KJ726426
**RZ599**	*Acronicta fragilis*	USA	KJ726384	KJ726388	KJ726388	KJ726394	KJ726394	KJ726402	KJ726408	–	KJ726425	KJ726427
**RZ616**	*Agriopodes fallax*	USA	–	KC819667	KC819685	KC819701	KC819718	KC819734	KC819749	KC819764	KC819781	KC819798
**RZ611**	*Acronicta superans*	USA	–	KJ726389	KJ726389	KJ726395	KJ726395	KJ726403	KJ726409	KJ726413	KJ726420	KJ726430
**RZ615**	*Acronicta grisea*	USA	–	KJ726390	KJ726390	KJ726396	KJ726396	KJ726404	KJ726410	KJ726414	KJ726421	KJ726431
**RZ612**	*Acronicta tritona*	USA	–	KJ726391	KJ726391	KJ726397	KJ726397	KJ726405	KJ726411	KJ726418	KJ726422	KJ726428
**RZ613**	*Acronicta vinnula*	USA	KJ726385	KJ726392	KJ726392	KJ726398	KJ726398	KJ726406	KJ726412	KJ726415	KJ726424	KJ726429

We also examined molecular variation in *Agriopodes fallax*, *Agriopodes geminata*, *Agriopodes tybo*, and more than 80 species of North American Acronictinae, including exemplars from all recognized genera and species groups, using the barcode region (658 bp) of COI gene (Hebert et al. 2003). DNA was extracted from one or two legs removed from a dried specimen and processed at the Canadian Centre for DNA Barcoding, Guelph, Ontario. DNA extraction, amplification and sequencing protocols for the Barcode of Life initiative are given in Hebert et al. (2003). Haplotypes of all barcode sequences were compared initially with phylograms constructed using the (Kimura 2-parameter) neighbor-joining method as implemented on the BOLD website (Ratnasingham and Hebert 2007).

*Phylogenetic analysis.* Data matrices (6407 bp total) were analysed by non-model-based (parsimony) with equal weighting and model-based evolutionary methods (Bayesian Inference, BI). Parsimony analyses used New Technology heuristic searches (consisted of Tree Fusion, Ratchet, Tree Drifting and Sectorial searches) implemented in the program TNT v1.1 (Goloboff et al. 2003). The analysis was run with default parameters applied until the most parsimonious tree was found 1000 times. BI analyses implemented using MrBayes v3.1 (Ronquist et al. 2005). Data sets were partitioned by gene region into eight partitions. For the model of sequence evolution, a GTR + Γ model was selected as the most appropriate model for each gene partition based on the Akaike Information Criterion using FindModel (http://www.hiv.lanl.gov/content/sequence/findmodel/findmodel.html). The Bayesian analyses were run for 5 million generations, with every 1000th generation sampled. Clade robustness was estimated by posterior probabilities (i.e., PP) in MrBayes. Convergence was determined when the average standard deviation of split frequencies went below 0.05 and the PSRF (Potential Scale Reduction Factor) approached 1, and both runs had properly converged to a stationary distribution after a burn-in stage (of 5,000 sampled generations).

*Immature stages.* Last instars of *Agriopodes fallax* from 10 km E of Indian Lakes, Hamilton Co., New York were compared with those of all North American genera of Acronictinae, most species of Nearctic *Acronicta* and images of both the European (Beck 1999) and Japanese (Sugi 1987) Acronictinae. Larvae and eggs of *Agriopodes tybo* were collected from Velvet Ash (*Fraxinus velutina* Torr., Oleaceae) near the American Museum of Natural History Southwestern Research Station, Cave Creek Canyon, Cochise Co., Arizona, and subsequently reared indoors on White Ash (*Fraxinus americana* L.) and Green Ash (*Fraxinus pennsylvanica* Marsh.).

## Results and discussion

**Figure 1.**
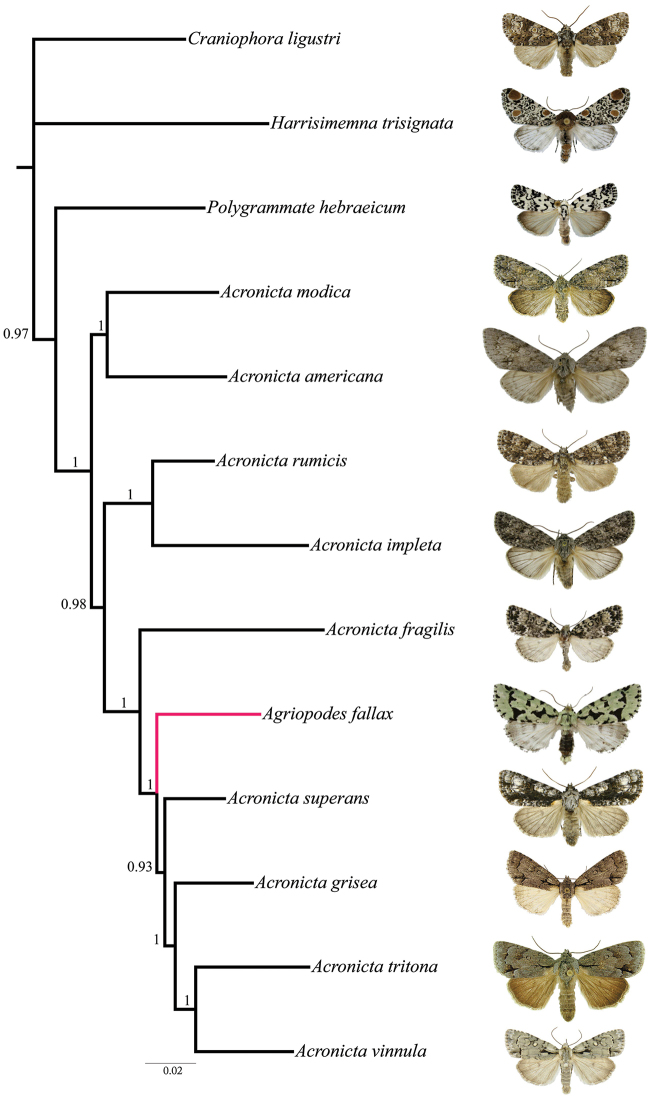
Phylogenetic placement of *Agriopodes fallax* (terminal branch in red) relative to 12 additional acronictine taxa, based on Bayesian analysis of eight gene regions. Bootstrap support values >50% are given for internal branches.

*Molecular analysis.* Phylogenetic analyses of the multi-gene dataset resolved a well-supported monophyletic *Acronicta* clade with the inclusion of *Acronicta fallax*, which placed as the sister species to a group consisting of *Acronicta superans* Guenée, *Acronicta grisea* Walker, *Acronicta tritona* (Hübner) and *Acronicta vinnula* (Grote) (Fig. 1). As discussed below, these four species represent two structurally delimited groups, with *grisea*, *tritona* and *vinnula* in the *tritona*-group and *superans* in the *hasta*-group (Schmidt and Anweiler unpubl. data).

Barcode variation showed that the single sample of *Agriopodes geminata* (641 bp) was very similar to haplotypes of *Agriopodes fallax* from ON, MI, FL, GA and OK, differing by 2–3 bp; comparison to barcode variation across *Agriopodes fallax* in the BOLD database (*n* = 25) indicated intraspecific variation of up to approximately 1.6% (Ratnasingham and Hebert 2007).

*Agriopodes tybo* barcode sequence showed little affinity to any sampled Acronictinae, differing by at least 8% from all other sequences in the BOLD database, which contains approximately 7300 species representing 250 genera of Noctuidae globally (BOLD). To explore a potential relationship of *Agriopodes tybo* to Amphipyrinae, Psaphidini, we compared *Agriopodes tybo* to a dataset constrained to Nearctic psaphidine genera (58 species and 29 genera), but minimum divergences were similarly upwards of 7%; as might be expected, nodes of intergeneric relationships were unsupported (data not shown). Barcode sequence of *Agriopodes tybo* was not found to be phylogenetically informative as to probable subfamily membership. The generic placement of *Agriopodes tybo* is currently the focus of an expanded study by Wagner et al. (in prep.).

### Acronicta fallax comb. n.

Taxon classificationAnimaliaLepidopteraNoctuidae

Figs 2 , 5 , 9 , 13

#### Adult morphology.

The fate of the genus *Agriopodes* is anchored to the phylogenetic position of *Acronicta fallax*, its type-species. Comparison of genitalic structure of *Acronicta fallax* to all North American and most Eurasian Acronictinae species reveals that genitalic features are most consistent with those found across an endemic North American group of *Acronicta* species, consisting of *Acronicta tritona*, *Acronicta grisea*, *Acronicta falcula* (Grote), *Acronicta lithospila* Grote, *Acronicta hamamelis* Guenée, *Acronicta mansueta* Smith, *Acronicta paralella* (Grote) and *Acronicta vinnula*, here termed the *tritona*-group. Structural synapomorphies for these species are primarily those of the male genitalia (Fig. 5), including a short, claw-like clasper and a broad shield-like juxta (wider than long), with strap-like dorso-lateral extensions. The male vesica structure is moderately complex and consists of a sausage-shaped main chamber that curves ventrally then right laterally, which is armed with short, spade- to thorn-like spines to longer attenuated spines. The size and position of the vesica diverticula are unique, with thumb-like diverticula consistently present in the basal and sub-basal positions, and smaller diverticula variably present in the medial and apical positions. In females, the corpus bursae is relatively broad and rounded, shaped like a heart or a boxing glove with the appendix bursae forming the ‘thumb’ (Fig. 9). Females of the *tritona*-group (Figs 10, 11) lack the dense, persistent patch of fine, felt-like hairs between the 8^th^ tergite and sternite that is present in the *Acronicta hasta*-group. The *hasta*-group contains at least 14 species, largely corresponding to “Group II” of Forbes (1954). As also suggested by the placement of *Acronicta superans* in our tree (Fig. 1), the *hasta*-group is related to the *tritona*-group, but exhibits a number of distinctive autapomorphies not present in either the *tritona*-group or *Acronicta fallax*, such as a unique hourglass-shaped juxta; modification of the quadrate ventral process of the clasper into a broad, scoop-like flange and a dorsally curved pollex; and as noted above, a patch of persistent felt-like setae on the female A8 pleuron.

Structurally, *Acronicta fallax* shows clear affinities to *Acronicta grisea* and *Acronicta falcula* of the *tritona*-group; the valve, clasper and uncus are much like those of *Acronicta grisea*, with the clasper apex slightly less curved. The dorsolateral straps of the juxta are spinulose, and the medioventral portion of the juxta is produced into a rounded knob that is unique to *Acronicta fallax*, although *Acronicta tritona* shows a rudimentary form of this. Aedeagus and vesica structure of *Acronicta fallax* are also similar to those of *Acronicta grisea* and *Acronicta falcula*, with two basal, unarmed diverticuli, a spinose main chamber, and a finely spinulose distal portion of the main chamber. The large spine field is composed of short, broad-based spines basally, and rounded, spade-like spines distally, similar to those found in *Acronicta tritona*. The female genitalic structure of *Acronicta fallax* is most similar to *Acronicta grisea* (Fig. 10). Larval morphology does not offer support for a special association among *Acronicta fallax* and *tritona* / *falcula* / *grisea*, although there is greater similarity of *fallax* to the *tritona* group than to larvae of the *hasta*-group.

Many *Acronicta* species bear a prominent black basal, anal and apical forewing dash; the basal and anal dashes are sometimes transected by a crescentic line resulting in a dagger-like mark (hence the common name dagger moths). These forewing dashes typical of *Acronicta* are also present in modified form in *Acronicta fallax*, with the apical and anal dash (dagger marks) broadly joined to the postmedial line to form two roughly triangular postmedial patches. The basal dash is short and thick; and there is a black rectangular bar connecting the orbicular and reniform spots; the orbicular and reniform spots are occasionally and then only incompletely outlined. Unlike the green psaphidines (Amphipyrinae, Psaphidini: *Feralia* Grote and *Miracavira* Franclemont), the green pigment of *Acronicta fallax* is not sensitive to moisture degradation, where green changes to yellow upon exposure to high humidity (dried specimens of *Acronicta fallax* can usually be moisture-relaxed without loss of green colouration). This suggests a fundamental biochemical difference in the green pigment of *Acronicta* (found in *Acronicta fallax* and *Acronicta vinnula*) compared to that of psaphidines.

**Figures 2–4.**
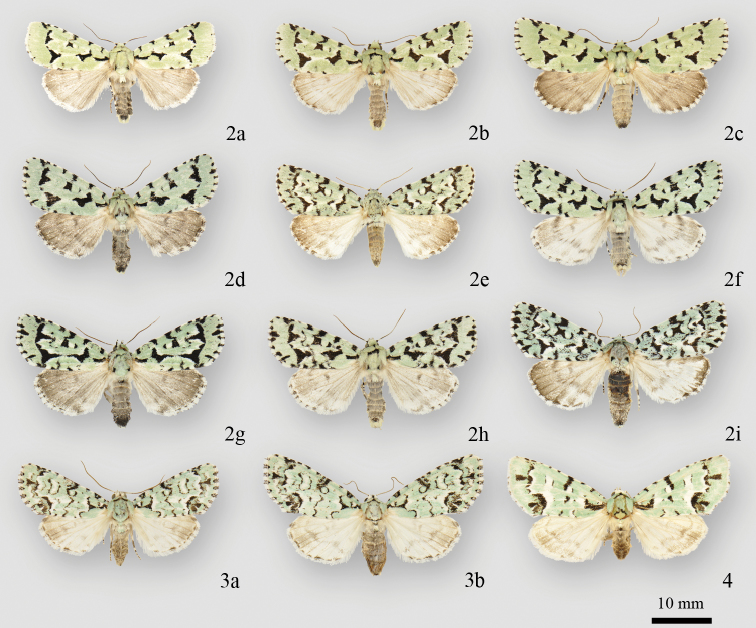
*Acronicta* and *Chloronycta* adults. **2a** *Acronicta fallax* male (North Port, FL) **2b** *Acronicta fallax* male (Hillsboro, MO) **2c** *Acronicta fallax* female (Hillsboro, MO) **2d** *Acronicta fallax* female (Backus Woods, ON) **2e** *Acronicta fallax* male (Cartwright, MB) **2f** *Acronicta fallax* male Edmunston, NB) **2g** *Acronicta fallax* female (Ottawa, ON) **2h** *Acronicta fallax* male (La Verendrye Reserve, QC) **2i** *Acronicta fallax* male (Crooked Lake, SK) **3a** *Chloronycta tybo* male (Huachuca Mtns, AZ) **3b** *Chloronycta tybo* female (Cave Ck. Cyn., Chiricahua Mtns, AZ) **4** *Chloronycta* sp. female (Turundeo, MEX).

**Figures 5–8.**
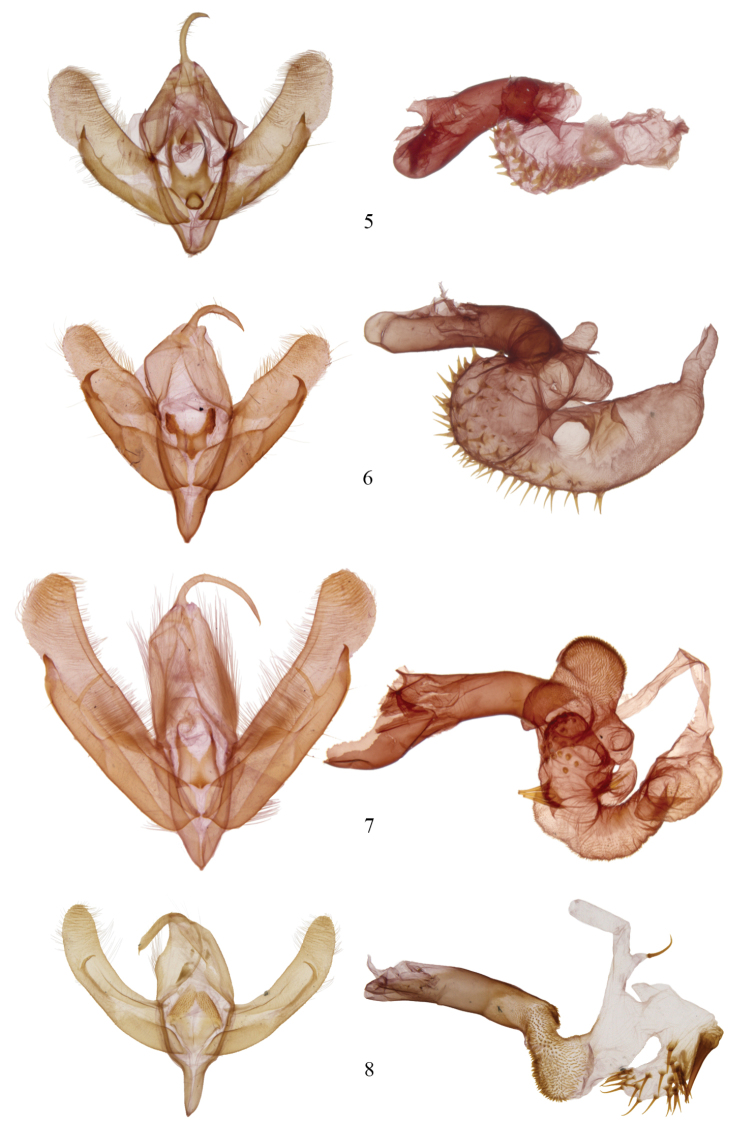
*Acronicta* and *Chloronycta* male genitalia. **5** *Acronicta fallax* **6** *Acronicta grisea* **7** *Acronicta tritona* **8** *Chloronycta tybo*. Reproduced to scale.

**Figures 9–12.**
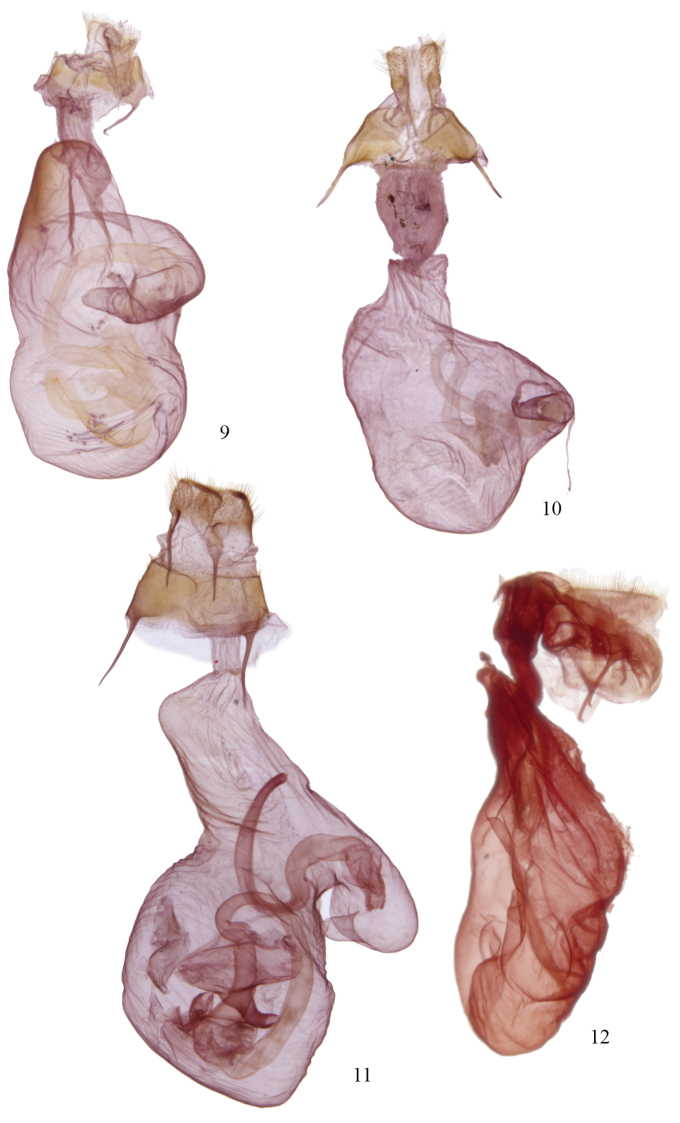
*Acronicta* and *Chloronycta* female genitalia. **9** *Acronicta fallax* **10** *Acronicta grisea* **11** *Acronicta tritona* **12** *Chloronycta tybo*.

#### Larval morphology and biology.

The immature stages of *Acronicta fallax* were described by Dyar (1899), and Crumb (1956) gives a brief description based on a preserved larva. Illustrations are in Wagner et al. (2011) and McCabe (1991; head capsule and mandible). Dyar described the waxy-white egg as a flattened dome with about 48 radial ridges lacking transverse striae, 2 mm across and 1 mm in height–characters consistent with those of *Acronicta*. *Agriopodes fallax* shares structural similarities with members of the *Acronicta hasta* and *Acronicta tritona* species groups; head often with 6–8 dark (snowflake-shaped) spots over each somewhat quadrangular lobe, D1 borne from small wart on T1, and the ground colour tends to be green and body height highest through the anterior abdominal segments in both clades. Superficially, *Acronicta fallax* shares greatest similarity with larvae of *Acronicta vinnula* and kin, a member of the *tritona*-group, although We cannot identify its sister taxon with certainty based on our knowledge of its morphology, behavior and natural history.

The mature larva is bright lime to yellowish green with a whitish middorsal and somewhat broader subdorsal stripe, with body tapering posteriorly. The integument is covered with abundant, minute secondary setae in the form of spinules that are slightly thickened basally, giving the integument a velvety texture. With the exception of the D1 pinaculum on T1, which is brownish to red and borne from a small wart, the pinacula are nearly obsolete in the last instar, i.e. flattened, faintly brown or concolourous, and with short setae (pinacula are more elevated and reddish brown with longer setae in middle instars). The greenish head sometimes has paired dark spots above the frons and a field of 6–8 darker spots over each lobe, laterad to apex of frons. The head, usually retracted into the thorax, has a rough, granular surface but lacks secondary setae, and is about 4 mm wide when mature. The thoracic shield is lightly sclerotized; prothorax with XD setae longest on body, extending well forward; XD1 and D1 solitary; D2 setal cluster shifted forward and grouping with XD2 seta; SD and L setae grouped, each comprised of 8–11 setae. Nearly all primary setae are replaced with open but defined clusters of 6–12 setae. Abdomen with D, SD, and L setal clusters more or less vertically aligned; D2 in typical position on A7–A10; solitary seta present below L2 group, well forward of spiracle; L3 group a diffuse set of 9–12 setae; numerous setae in each subventral cluster. A8 spiracle approximately 2 × diameter of those on preceding segments. The anal plate and pinacula are ill defined or undifferentiated, with limits defined by clusters of microspinules, which are largest (some tooth-like) over the anal plate. Prolegs with 23–28 crochets. Length of larva at maturity is 28–30 mm. The prepupal larva turns waxy red, and tunnels into soft wood or spins a flimsy cocoon in a crevice. The larva feeds from the leaf underside of *Viburnum* species, including *Viburnum dentatum* L. (Dyar 1899) and *Viburnum nudum cassinoides* (L.) Torr. & A. Gray (Wagner et al. 2011). Undoubtedly, other *Viburnum* species are used also, particularly by northern populations beyond the range of *Viburnum dentatum* and *Viburnum nudum*. A record for poplars (*Populus* sp.) as a host cited by Tietz (1972) is certainly erroneous.

**Figures 13–16.**
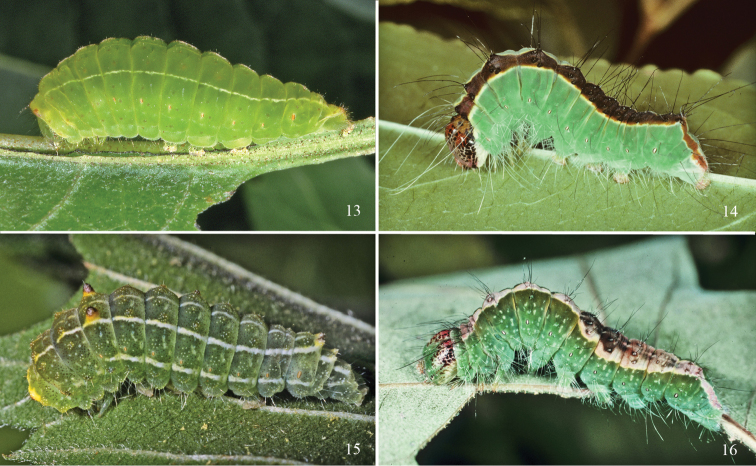
*Acronicta* last instar larvae. **13** *Acronicta fallax* (Norfolk, CT) **14** *Acronicta superans* (Norfolk, CT) **15** *Acronicta vinnula* (Coventry, CT) **16** *Acronicta lithospila* (Martha’s Vineyard, MA).

### Agriopodes geminata

Taxon classificationAnimaliaLepidopteraNoctuidae

(Smith)

Moma geminata Smith, 1903

#### Remarks.

*Acronicta fallax* exhibits a moderate range of geographic variation (Fig. 2), with a gradual increase in size and extent of forewing black markings with increasing latitude. The northwestern-most populations from Manitoba, Saskatchewan and eastern Alberta mark the extreme end of this cline, and have been treated as a separate species, *Agriopodes geminata*. We can find no consistent differences in genitalic structure between *Acronicta fallax* and *Acronicta geminata*, and differences in COI barcodes fall well within the range of variation of *Acronicta fallax*, as discussed above. We therefore consider *geminata* to be a geographic form of *Acronicta fallax* (syn. n.). *Acronicta geminata* Draudt, 1950 of China is a junior secondary homonym of *Acronicta geminata* (Smith), but since we treat the latter as a junior subjective synonym of *Acronicta fallax*, no replacement name for *Acronicta geminata* Draudt is necessary.

### Agriopodes corticosa

Taxon classificationAnimaliaLepidopteraNoctuidae

(Guenée)

Bryophila corticosa Guenée, 1852

#### Remarks.

The identity of this taxon remains an enigma. We have been unsuccessful in locating type specimens in collections housing Guenée types (The Natural History Museum, London; National Museum of Natural History, Washington, D.C. ), and the specimens are presumed to be lost. No illustration accompanied the description, a translation of which follows (our comments in square brackets):

“Same size as [*Noctua*] *Glandifera* [a junior subjective synonym of *Nyctobrya muralis* (Forster, 1771; Bryophilinae), diagnosed by Guenée in the account previous to *corticosa*]. Forewings broad, scaly, variably coloured with greenish white, light brown and black, and with all of the lines black. Basal space also greenish white, with the basal line and one spot at the costal border black. Median space of a grey brown, with the disc [claviform spot] lightly dusted with a fawn color; terminal space spotted with brown. The subterminal line very dark black, very undulated, and more or less parallel to the postmedial line. Fringe whitish, streaked with many fine black lines. Hindwings pearly white, with a blackish shade at the base of the interior [anal] angle and extending nearly halfway along the wing. Underside of the abdomen white. Body rather big. Antennae long.

North America. Boisduval collection. Two specimens.”

Since its description, the identity of this taxon has been uncertain, and it has often been omitted entirely (e.g., Hampson 1908, 1909). Guenée originally associated it with *Bryophila* Treitschke, and nearly all of Guenée’s *Bryophila* species are currently placed in Bryophilinae (Fibiger et al. 2009), although three Nearctic “*Bryophila*” species are now placed in Noctuinae, Xylenini (*Chytonix palliatricula* (Guenée)) and Acronictinae (*Cerma cora* Hübner and *Polygrammate hebraeicum* Hübner) (Lafontaine and Schmidt 2010). Dyar (1902) listed it as *Jaspidia corticosa* (Guenée) along with species now in *Leuconycta*, *Cryphia*, and *Anterastria*. Hampson (1909) established *Agriopodes* but overlooked *corticosa*, and Barnes and McDunnough (1917) subsequently expanded Hampson’s concept of *Agriopodes* to include *corticosa* for reasons that are unclear, except perhaps that they felt the original description of *corticosa* rendered it closest to *Agriopodes fallax*. The placement of *corticosa* in *Agriopodes* was maintained by Poole (1989). Hodges et al. (1983: p. ix) excluded *Agriopodes corticosa* from the North American fauna based on an assessment that it most likely represented an Asian or South American species.

The description, comparison to *Nyctobrya muralis* (Forster) (see e.g., Fibiger et al. 2009: pl.12 Figs 42–48), and inclusion of other externally similar species (primarily European Bryophilinae) in Guenée’s concept of *Bryophila*, leave little doubt that *corticosa* is a greenish-white and black noctuid, probably with a lichen-mimicking pattern (i.e., one that occurs uncommonly but repeatedly in unrelated noctuid lineages). If one assumes the North American origin of the type as correct, there are only a few species that *corticosa* could possibly refer to. All of the approximately 200 North American taxa named by Guenée during this time occur in eastern North America, and none is strictly western, although Boisduval, the source of the *corticosa* types, was describing Californian species at this time. Eastern North American noctuids with pale green forewing patterning include *Feralia* (three species), *Leuconycta* (two species), and *Acronicta fallax*. Guenée named and described *Feralia jocosa* under a different family seven pages after *corticosa*, so in addition to the differences in *Feralia* facies to Guenée’s *corticosa* description, *Feralia* can safely be ruled out as a candidate for the identity of *corticosa*. Similarly, *Leuconycta diphteroides* (Guenée) was described by Guenée four pages after *corticosa*, so this species, and the externally similar *Leuconycta lepidula* (Grote), are unlikely candidates for *corticosa*. This leaves the possibility of *Agriopodes fallax* as the true identity of *corticosa*, an interesting prospect since *corticosa* would be the senior name. A number of points in Guenée’s description of *corticosa*, however, cannot be construed as *Agriopodes fallax* characters, most notably the mention of brown colouration in the forewing, presence of a claviform spot, an undulating postmedial line, a white hindwing with dark scaling only at the anal margin, and a smaller wingspan. Expanding the possibilities for the identity of *corticosa* to western North American species likewise provides no further leads; the Nearctic *Cryphia* (endemic to the West) and southwestern *Bryophila* do not match Guenée’s description. No European species easily fit the description either, and Guenée gave a diagnosis of all other European Bryophilinae known at that time, so he would have recognized them as such, or at least compared *corticosa* to other European species. The only thing that seems certain is that *Bryophila corticosa* is not identifiable as a European or North American species, as Franclemont and Todd [1983] also concluded, and there is no justification for maintaining it as a species of *Agriopodes*. We therefore consider *Bryophila corticosa*, comb. rev., a *nomen dubium* that cannot be placed in any noctuid subfamily.

### Fate of *Agriopodes*

The handsome green-mottled forewing colour and pattern of *Agriopodes* have resulted in a century of erroneous systematic placement of the eight species included at one time or another in this genus. Despite the remarkable divergence in wing pattern from other *Acronicta* species, our genitalic, external morphological, larval, and molecular character evidence confidently places *Agriopodes fallax* as an *Acronicta*.

The taxonomic fate of *Agriopodes* is complicated somewhat by the broad scope of the genus *Acronicta*. Beck (1999) took the extreme approach of splitting 13 European *Acronicta* species into nine genera, whereas a conservative approach of a single genus with six subgenera was subsequently proposed by Fibiger et al. (2009). Generic or subgeneric division for the North American *Acronicta* has not been proposed but an expanded concept of the genus is generally consistent with the view of Forbes (1954), who recognized five informal species-groups within *Acronicta*. A subgeneric classification similar to the divisions proposed by Fibiger et al. (2009) is being developed for the Nearctic, and reconciled with the Eurasian fauna (Schmidt and Anweiler, in prep.). We do not formally propose *Agriopodes* as a subgenus here, as it requires addressing the remaining genus-group names and more than 80 North American species of *Acronicta*.

### Chloronycta

Taxon classificationAnimaliaLepidopteraNoctuidae

Schmidt & Anweiler gen. n.

http://zoobank.org/D57F9ACB-5959-4966-BC64-E3D754B69BBE

#### Gender.

Feminine.

#### Type species.

*Moma tybo* Barnes, 1904.

#### Diagnosis.

Two species are included in *Chloronycta*, *Chloronycta tybo* and an undescribed Mexican species near *Chloronycta tybo*, known to us from only one female specimen (Fig. 4) and therefore it is not formally described here. The only Nearctic species externally similar to *Chloronycta* is *Acronicta fallax*, but *Chloronycta* lacks the black bar between the reniform and orbicular spots, has the reniform and orbicular stigma finely outlined in black, and has various black markings in the subterminal space, which is entirely green in *Agriopodes fallax*. The two genera do not overlap in range, with *Chloronycta* essentially a Mexican taxon reaching southeastern Arizona, and *Agriopodes fallax* restricted to deciduous forests of eastern North America. Genitalic structure of the two genera is very different (Figs 5, 8, 9, 12). The main diagnostic characters for *Chloronycta* are 1) forewing ground colour pale bluish green and white, the green colouration not degrading to yellowish with exposure to moisture; 2) valve apex with flattened, corona-like setae; 3) vesica with a single long spine isolated at the base of the ductus ejaculatorius; 4) tympanal sclerite consisting of rounded, adjoining nodules, not flange- or scoop-like.

#### Description.

Head. Antenna of male simple-prismatic, such that ventral margin appears slightly serrate when viewed laterally, evenly ciliate laterally and ventrally; female antenna similar but with segments less produced ventrally; antenna with dorsal scales grey, grading to white scales over basal third, with scattered black scales; haustellum normal, approximately equal in length to that of thorax; eye smooth, round; labial palpus with 3^rd^ segment 0.4 × length of 2^nd^ segment; 1^st^ segment clothed with black spatulate scales dorsally and longer, strap-like white scales ventrally; 2^nd^ segment with short, spatulate white scales apically and basally and with black scales forming broad, dark band medially; 3^rd^ segment with short spatulate white scales and scattered black scales; frons with short, appressed, spatulate scales and longer strap-like scales forming a medial crest near ventral margin; scales of frons white, except for a patch of black scales medio-laterally; occiput with longer spatulate white scales, with black scales forming a black medial crest-like line. Thorax. Prothoracic collar with pale bluish-green spatulate scales, bordered dorsally and along eye margin by narrower black scales; mesothorax, metathorax and tegula clothed in bluish-green spatulate scales, margin of tegula with longer hair-like scales; mesothorax with paired patch of subdorsal black scales at posterior margin; tympanal sclerite raised, rounded and spade-like; prothoracic leg with brown-black and white scaling, femur brown black dorsally and greyish white ventrally; tibia black, with a transverse medial and apical band of white scales; epiphysis 0.5 × length of tibia; tarsal segments black scaled, with a distal band of white scales; scaling pattern of meso- and metathoracic legs similar to that of prothoracic leg; tibial spines white scaled, tibial spine formula 0-2-4. Abdomen. Clothed in a mix of brownish-grey and whitish scales, which are short, spatulate and closely appressed; A1 with a dorsal tuft of long spatulate, bluish-green scales; terminal scales white and hair-like; A8 sternites and tergites normal. Male genitalia (Fig. 8). Uncus rod-like, about 5–6 × as long as wide, with a short, curved terminal spine; tegumen roughly rhomboid and broad, 2.3 × longer than wide; vinculum with saccus well developed, base slightly constricted, 2.5 × longer than base width; valves relatively simple, straplike and parallel sided, about 4.5 × longer than wide, evenly curved in a shallow arc; sacculus moderately developed, clasper well developed but thin, slightly curved with a sharp terminus, located 4/5 distance to valve apex, ampulla absent; area between clasper and terminus covered in long fine setae, setae on outer 2/3 thicker and flattened; setae near margin of apex more robust and lance-like, directed towards base of valve; juxta well sclerotized, spinose and rasp-like dorsolaterally, dorsal margin divided; aedeagus 4.5 × longer than wide, nearly straight, slightly decurved ventrally; basal half of vesica angled slightly downward, densely clothed with small but prominent spines, curving dorsad and expanding into a large medioventral diverticulum, this about as wide as long, with 15–20 long prominent spines and two to three massive, partially fused spines at base; vesica narrows abruptly beyond diverticulum, extending anteriorly, poorly differentiated from ductus ejaculatorius; a single prominent, elongate, curved spine arising from small narrow pouch near vesica terminus / base of ductus. Female genitalia (Fig. 12). Corpus bursae elongate globose, 1.25 × longer than wide, with invaginated sclerotized area dorsally at base of ductus bursae; appendix bursae dorsal and to to right of ductus bursae, small and indistinct, tapering abruptly to ductus seminalis; ductus bursae membranous, rugose, 1.5 × longer than wide; ostium bursae moderately sclerotized, with v-shaped ventral notch; antevaginal plate somewhat sclerotized and covered with dense, minute setae, projecting caudad as a somewhat pointed scoop; apophyses short, posterior apophysis 0.8 × and anterior apophysis 0.7 × height of papillae; papillae anales densely setose, margin quadrangular with slightly protuberant ventrocaudal angle.

#### Biology and distribution.

*Chloronycta* occurs in the mountainous regions from Mexico to south-eastern Arizona and southwestern New Mexico, where it reaches the northern terminus of its core range in the Sierra Madre Occidental. *Chloronycta tybo* occurs in canyons and mid-elevation wooded habitats, particularly riparian corridors where the larval host plant, *Fraxinus velutina*, grows. The larva and host plant of *Chloronycta tybo* are described here for the first time. The larval description under *Agriopodes tybo* in Powell and Opler (2009), based on an account by J. A. Comstock, actually refers to Comstock’s (1957) description of *Agriopodes viridata* (Harvey), now placed in the genus *Bryolymnia* (Noctuinae, Elaphriini; Lafontaine et al. 2010).

**Figures 17–20.**
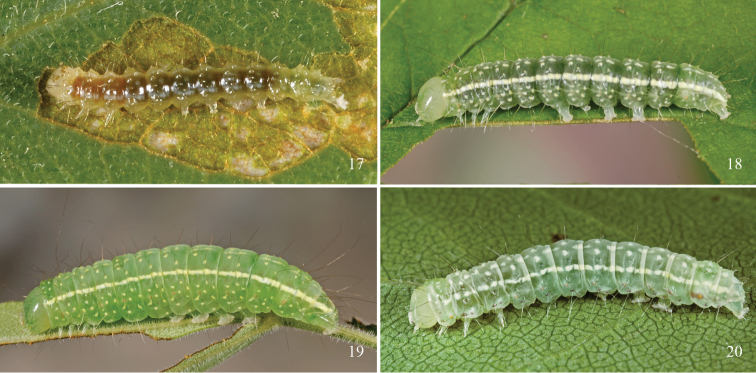
*Chloronycta tybo* (Cave Creek Canyon, AZ) and *Polygrammate hebraeicum* (Cosby, TN) larvae. **17** *Chloronycta tybo* early instar **18** *Chloronycta tybo* penultimate instar **19** *Chloronycta tybo* ultimate instar **20** *Polygrammate hebraeicum* ultimate instar. In addition to overall resemblance, note similarities in dorsal pattern elements, as well as head allometry, colouration, and luster (particularly between penultimate instar of *Chloronycta tybo* and ultimate instar *Polygrammate hebraeicum*).

The first two larval instars (Fig. 17) are leaf skeletonizers that remove patches of leaf tissue from the lower leaf surface. Middle (Fig. 18) and late instars feed from a leaf edge, always from the underside of a blade.

Ultimate instar larva (Fig. 19) (total length to 26 mm, *n* = 3) waxy green, integument translucent, body thickest through A3–A5, strongly tapered rearward. Broken middorsal stripe composed of single lines on T2, T3, and A9, and anterior and posterior lines on A1–A8; broad, creamy subdorsal stripe that gradually widens posteriorly, extending from T1 through to and including anal plate. D1 and D2 pinacula free on all segments. Primary setae only, these fine; D, SD, L, and SV group setae borne from cream-yellow, pimple-like pinacula embedded within a pale yellow spot; longest setae black. One additional yellow subventral spot on T1–T3; four additional yellow lateral spots on A1–A8. D2 on T2 extending well forward of head. D2 on all abdominal segments 1.5 × longer than D1. SD1 and L2 very long on abdominal segments, circa 2 × length of an abdominal segment. D2 setae on A9 and A10 elongate, trailing behind body. Prolegs with 32–35 crochets. Spiracles tan yellow with brown peritreme. Entire integument microspinulose. Head immaculate pale green, shiny, translucent; labrum creamy. Prepupal larva flushed with red.

Prepupae tunnel into punky wood when available to form a pupal crypt, largely free of silk, with the exception of that used to weave the frass-silk cover that renders the pupal chamber essentially invisible to the untrained eye.

#### Remarks.

Few acronictine groups have appreciable Neotropical representation (there are no confirmed South American Acronictinae). The majority of species are temperate and cold temperate; one exception in the New World is the *Acronicta theodora* Schaus group, which reaches Costa Rica. Acronictine genera are most diverse in temperate Asia but many genera, subgenera and species-groups are shared between the two realms (e.g. *Harrisimemna* Grote, *Simyra* Ochsenheimer and *Acronicta* subgenera *Acronicta*, *Jocheara* Hübner, and *Hyboma* Hübner). *Chloronycta* may also be derived from an Asian ancestral group, although there are admittedly no obvious sister taxa—*Moma* Hübner and *Nacna* Fletcher are similar in facies, but neither belong to the Acronictinae (Wagner et al., in prep).

Despite the very similar forewing colouration of *Chloronycta* to *Acronicta fallax* the two share no uniquely derived structural traits; DNA barcode sequence also does not support an association between with these taxa. Genitalic structure in *Chloronycta* is unique among Acronictinae, and no close relatives are evident. The simple valve structure, short saccular process along the ventral valve margin, and weakly-developed corona are shared by the Asian acronictine genus *Subleuconycta* Kozhanchikov (Kononenko and Han 2007). Larvae of this Old World genus are not known.

As there are no unequivocal adult or larval autapomorphies for Acronictinae (Kitching and Rawlins 1998; Wagner 2007a, b), we also examined the possibility of *Chloronycta tybo* belonging to other basal trifine subfamilies, most notably the Amphipyrinae, Psaphidini, which share some similarities with *Chloronycta tybo* (elongate valve, flattened setae forming valve corona, green forewing colouration, elongate tegumen). As is the case for Acronictinae (Beck 1999; Wagner 2007a, b), the best diagnostic characters for Psaphidini are those of the immature stages (Wagner et al. 2008). The larva of *Chloronycta tybo* shares a number of similarities with those of *Comachara* Franclemont and *Polygrammate* Hübner (Fig. 20; Wagner et al. 2011); all three genera share a similar ground colour; long, slender, darkened dorsal setae; proportionately large, pale green, smooth and unmarked head capsule; thickened subdorsal line and broken middorsal stripe represented by anterior and posterior fragments. Most significantly among the shared features, the first two sets of prolegs are somewhat reduced in size in all three genera. However, the absence of secondary setae immediately differentiates *Chloronycta tybo* from all other North American Acronictinae except *Cerma*. Behavioral characters linking *Chloronycta tybo* to many Acronictinae include the wood-tunneling habits of the prepupae; mature larvae excavate pupal chambers in punky wood, then seal the entrance with a sheeting of silk and frass in the same fashion as various acronictine genera. However, while tunneling into wood, *Chloronycta tybo* larvae do not roll the frass shavings into balls as do some basal members of the Acronictinae (e. g., *Comachara*, *Harrisimemna*, and *Polygrammate*) (Wagner 2007a, b). *Chloronycta tybo* turns reddish as a prepupa, as do nearly all wood-tunneling Acronictinae.

The following adult characters associate *Chloronycta* with the Acronictinae: 1) a black ‘eye-stripe,’ which in the natural resting position of the moth is formed by black scaling on the middle of labial palpus segment 2, on the prothoracic collar behind the eye, and extending into the basal dash of the forewing; 2) dorsal tuft of scales on A1 (occurring also in unrelated subfamilies such as Plusiinae, but absent in Psaphidini); 3) legs strongly banded in black and white, shared with other acronictines including *Polygrammate*, *Harrisimemna*, and *Cerma* (Wagner 2007a, b), but absent in most Amphipyrinae, Psaphidini (present in *Feralia* and *Miracavira*); 4) flattened, dome-shaped eggs; and 5) Oleaceae as a larval host plant family. The use of *Fraxinus* is very rare among noctuids, only six other North American species (none acronictines) are known to do so (Wagner 2007; Wagner, in prep.). Yet, a number of Asian acronictine genera (*Craniophora* Snellen, *Acronicta* subgenus *Plataplecta* Butler, *Thalatha* Walker, *Thalathoides* Holloway) specialize on this family (Holloway 1989), suggesting and ancient link between *Chloronycta* and the diverse Oleaceae-feeding genera of eastern Asia.

The discovery of a weakly-developed valval corona is noteworthy as all Acronictinae were previously thought to lack this structure (Fibiger and Lafontaine 2005; Lafontaine and Fibiger 2006). If placed correctly in the Acronictinae, the Asian *Subleuconycta* would provide a further example of a corona (Kononenko and Han 2007) in the dagger subfamily. The confirmed presence of this male genitalic trait considerably strengthens the hypothesis that Acronictinae are related to the Amphipyrinae-group of noctuid subfamilies, and that the presence of secondary larval setae, shared with the more basal Pantheinae lineages, is homoplasious (Zahiri et al. 2013b).

## Conclusions

Examination of morphological and molecular data shows that *Acronicta fallax* is phylogenetically rooted to the *Acronicta tritona*/*hasta* groups, and that *Acronicta fallax* and *Acronicta geminata* are conspecific. Accordingly, we place *Agriopodes* within the current concept of *Acronicta* as a synonym. In contrast, *Chloronycta tybo* is not closely related to *Acronicta fallax*, *Acronicta*, or, evidently, any other genus of Acronictinae, although available evidence places it in the subfamily in the vicinity of basal genera such as *Polygrammate*. Additional morphological and molecular studies with greater taxon sampling are needed to determine its phylogenetic position within basal noctuids. *Bryophila corticosa*, previously included in *Agriopodes*, cannot currently be associated with any known Noctuidae species (or subfamily), and remains a *nomen dubium*. The following nomenclatural changes are proposed:

***Acronicta* Ochsenheimer**

*Agriopodes* Hampson, 1908, *Catalogue of the Lepidoptera Phalaenae in the British Museum* 7: 16. **syn. n.**

**Type species.** *Moma fallax* Herrich-Schäffer, 1854, by subsequent designation by Hampson 1909, *ibidem*, 8: 37.

***Acronicta fallax* (Herrich-Schäffer), comb. n.**

*Diphthera fallax* Herrich-Schäffer, [1854], Sammlung neuer oder wenig bekannter aussereuropäische Schmetterlinge **1:** pl. 42, f. 211, wrapper.

**Type locality.** Tennessee, [USA]. [types lost]

*Moma geminata* Smith, 1903, Journal of the New York Entomological Society 11: 1. **syn. n.**

**Type locality.** Cartwright, Manitoba [Canada]. [American Museum of Natural History, New York]

***Chloronycta* Schmidt & Anweiler, gen. n.**

**Type species.** *Moma tybo* Barnes, 1904

***Chloronycta tybo* (Barnes, 1904), comb. n.**

*Moma tybo* Barnes, 1904, Canadian Entomologist, 36: 166.

**Type locality.** Cochise Co., Arizona. [National Museum of Natural History, Washington, D.C.]

Within the Noctuoidea, green and black lichen patterning has arisen at least nine separate times in North American taxa: *Afrida* Möschler (Nolidae), *Acronicta* (Acronictinae), *Bryolymnia* and “*Elaphria*” *cyanympha* (Noctuinae, Elaphriini), *Leuconycta* (Condicinae), *Cryphia* (Bryophilinae) and *Feralia*, *Miracavira*, *Emarginea* Guenée (Amphipyrinae, Psaphidini). There are likely also multiple independent derivations in the biochemistry of green-scale pigmentation, since green pigments are moisture sensitive in, for example, psaphidines and geometrines (Geometridae), but not in acronictines. The green pigment may be a novel autapomorphy for the Acronictinae, parallel to the geoverdin pigment present in Geometridae, Geometrinae (Cook et al. 1994).

**Figure 21.**
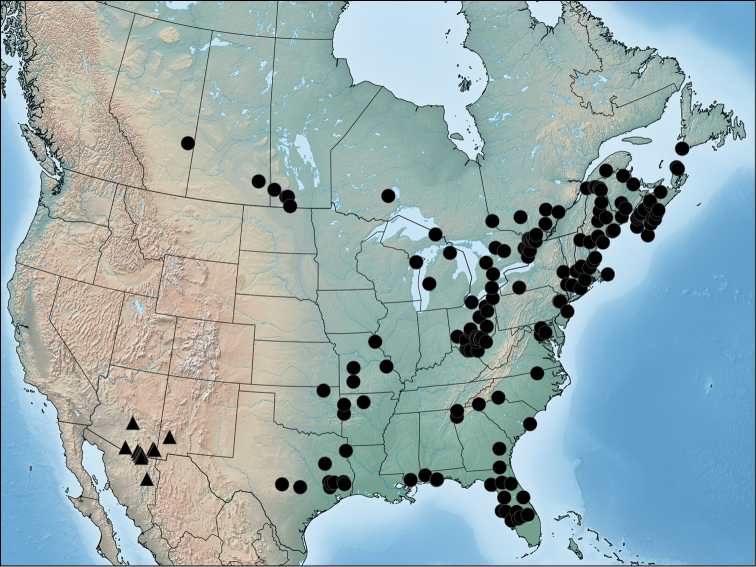
Distribution of *Acronicta fallax* (circles) and *Chloronycta tybo* (triangles) based on material examined in this study.

## Supplementary Material

XML Treatment for Acronicta fallax

XML Treatment for Agriopodes geminata

XML Treatment for Agriopodes corticosa

XML Treatment for Chloronycta
